# Introduction of the community first responder system into Japan: is that possible?

**DOI:** 10.1186/1865-1380-6-34

**Published:** 2013-09-30

**Authors:** Yoshiki Toyokuni, Masayuki Suzukawa, Keisuke Yamashita, Chikara Yonekawa, Katsuaki Kubota, Yasuharu Yasuda, Akihiro Kobayashi, Hiroki Matsubara

**Affiliations:** 1 Department of Emergency and Critical Care Medicine, Jichi Medical University, Tochigi, Japan; 2 National Research Institute of Fire and Disaster of Japan, Tokyo, Japan; 3 Department of Clinical Engineering, Hiroshima International University, Hiroshima, Japan; 4 Haga Fire Department, Tochigi, Japan; 5 Kaga Fire Department, Ishikawa, Japan

**Keywords:** Out-of-hospital cardiac arrest (OHCA), Community first responder (CFR), Emergency medical service (EMS), Japan

## Abstract

**Background:**

To improve out-of-hospital cardiac arrest (OHCA) survival rates in Japan, implementation of a community first responder (CFR) system is considered one of the most effective emergency medical service options. We investigated the possibility of introducing a CFR system in Japan.

**Methods:**

Cross-sectional surveys were given to 1,350 residents over the age of 18 who were selected from resident registration lists in Tochigi prefecture. Residents were questioned whether they would agree to have a CFR system in their community and whether they would participate as a responder. Positive attitudes about the cross-sectional study led us to conduct pilot CFR trials. Trials were conducted in rural areas of Tochigi prefecture by local EMS personnel. We were able to discuss and develop CFR introduction guidelines for Japanese communities using the results of the individual surveys, pilot trials, and other countries’ guidelines. Finally, our CFR system, which referred to developed CFR introduction guidelines, was introduced into Ishikawa prefecture’s Shioya town (population of 710).

**Results:**

A total of 92.5% of Tochigi residents either strongly agreed or agreed to have a CFR system in their community, and 16.7% of Tochigi’s residents chose to participate. The two CFR introduction prerequisites were identified as: (1) an information delivery system for CFR and (2) budget preparation. CFR introduction guidelines were developed, and a CFR system was introduced in Shioya town on 4 November 2012 with 32 participants. On 1 January 2013, a CFR responded for the first time, and the CFR system worked efficiently.

**Conclusions:**

By providing information about the CFR system to the community and preparing several infrastructural elements, it was possible to introduce and operate a successful CFR system in Japan.

## Background

Early resuscitation and defibrillation improve the survival rate of patients who experience out of hospital cardiac arrests (OHCA) [1]. Ambulance crews cannot achieve early patient care in areas that are not easily reached by an ambulance. In several remote areas of Australia, Europe and the USA, there are community first responder (CFR) systems. In the CFR system, local volunteers are dispatched by the local emergency medical service (EMS) to provide early cardiopulmonary resuscitation (CPR) and automated external defibrillator (AED) use before ambulance crews arrive. Several studies have showed that CFR systems improve the survival rate of OHCA [2,3]. In Japan, there are many remote areas and isolated islands, but no CFR operations. According to the Japanese resuscitation registry, the Japanese OHCA [cardiac origin, witnessed, ventricular fibrillation (VF)] survival rate was 20.8% in 2012 [4], while it was 46.3% in Rochester, Minnesota [5] (cardiac origin, witnessed, VF). Introduction of a CFR system into Japan’s remote areas could be one of the most effective EMS options to improve the Japanese OHCA survival rates in these areas. Therefore, in this study, we investigated the possibility of introducing the CFR system into remote areas in Japan and also investigated its preparatory requirements.

## Methods

We conducted individual surveys to identify Japanese people’s perceptions about the introduction of a CFR system. We also conducted CFR system pilot trials to identify any prerequisites that should be prepared during the actual introduction. Each requirement was then investigated. Finally, we developed introduction guidelines and introduced a CFR system into one selected remote area to test our guideline’s efficacy in introducing a successful CFR system to our country.

### Individual surveys

#### Study design

Cross-sectional surveys to identify Japanese people’s perceptions about the introduction of a CFR system were carried out in December 2011. Our research protocol to undertake individual surveys was approved by the Ethics Committee of Jichi Medical University (Shimotsuke, Tochigi, Japan) on 24 November 2011 (ethics approval no. 11-31). Informed consent was obtained from each subject by submission of a questionnaire sheet. Subject anonymity was preserved throughout the survey. No incentive was offered.

### Subject

Tochigi (population; 2,000,021) prefecture’s 1,350 residents over the age of 18 were selected from resident registration lists using two-stage stratified random sampling. The Tochigi prefecture includes Oyama, Mooka, and Motegi municipalities, which are considered rural areas.

### Data collection

A member of the survey team visited each individual subject’s home. An interviewer explained the details of the CFR system using slides to clarify any ambiguities before starting the survey questions. We asked about their willingness to integrate a CFR system into their communities. If a resident rejected the idea of incorporating a CFR system into their community, they were asked to specify why.

Each subject’s perceptions about participation as a CFR member were questioned and investigated. Those who said that they would not participate as a CFR member were asked for the reasons why they would not participate. We used these reasons to discuss how to increase participation. We also asked what the participants thought were the best communication devices for receiving requests from a dispatch center. This information was later used as a reference for developing information delivery systems at dispatch centers.

### Identifying preparatory requirements

#### Study design

Our cross-sectional survey revealed that Japanese people have a positive attitude toward the introduction of a CFR system in their community and also found that there are individuals who are willing to participate as a CFR. Therefore, to move on to the next step for introducing the CFR system into Japan, we decided to conduct CFR system trials to identify prerequisites that should be met for the actual introduction of a CFR system. Trials were conducted at Tochigi prefecture’s selected area of the Mooka and Motegi municipalities. Ambulances in this area take longer than 10 min to arrive after a 119 emergency call to a dispatch center.

#### Study protocol

Off-duty local EMS personnel acted as patients, CFRs, and ambulance crews. Trials started with a 119 emergency call. CFRs were dispatched, and upon arrival they performed CPR with the use of an AED. They ended their tasks by transferring patient care to the ambulance crew. Two simulations were carried out at the Motegi municipality, and three simulations were carried at the Mooka municipality. The possible preparatory requirements that we had anticipated were (1) CFR’s triage protocol, (2) an information delivery system for CFRs, (3) budget preparation, and (4) practical training for responding. After identifying preparatory requirements, we investigated each requirement individually.

### Development of CFR system introduction guidelines

CFR system introduction guidelines were developed using the results of individual surveys, trials, and other countries’ guidelines [6-9].

### CFR system introduction into a pilot area

Ishikawa prefecture’s Shioya town (population of 710) was chosen as the pilot area because the community residents understood the need for CFRs and were very willing to have a CFR program introduced. Also, the residents had experience in providing first aid from their town’s periodic disaster management training. Referring to our guidelines, they developed their own CFR operation system in cooperation with the local EMS and dispatch center. The possibilities of the introduction of a CFR system into the town and CFR system operations were observed.

## Results

### Individual surveys

A total of 60.4% (816/1350) of community members completed the survey. Concerning acceptance of a CFR system, 92.5% of Tochigi residents strongly agreed or agreed to have a CFR system in their community (Table 1).

**Table 1:** Individual survey results

**Prefecture**	**Municipality**	***N***	**Strongly agree/agree *N *(%)**	**Fair/disagree/strongly disagree *N *(%)**
Tochigi	Total of 3 municipalities	816	755 (92.5)	61 (7.5)
	Oyama	232	218 (94.0)	14 (6.0)
	Mooka	286	255 (89.2)	31 (10.8)
	Motegi	298	282 (94.6)	16 (5.4)

Response to CFR system introduction in own community.

Concerning the rejection of a CFR system, only four people disagreed with having a CFR system in their community at Tochigi. Their reasons for disagreement were: “It could be a good thing at the beginning but it may burn out after a while”. “Not interested”. “Too much work load for the responder”. “I believe there is more demerit than merit”.

Table 2 shows the results for the CFR participation rate. A total of 16.7% of Tochigi’s residents chose to participate.

**Table 2:** Individual survey results

**Prefecture**	**Municipality**	***N***	**Definitely yes *N *(%)**	**Somehow yes/do not know/no/definitely no N (%)**
Tochigi	Total of 3 municipalities	816	136 (16.7)	680 (83.3)
	Oyama	232	36 (15.5)	196 (84.5)
	Mooka	286	40 (14.0)	246 (86.0)
	Motegi	298	60 (20.1)	238 (79.8)

Response to participate in CFR.

**Figure 1:**
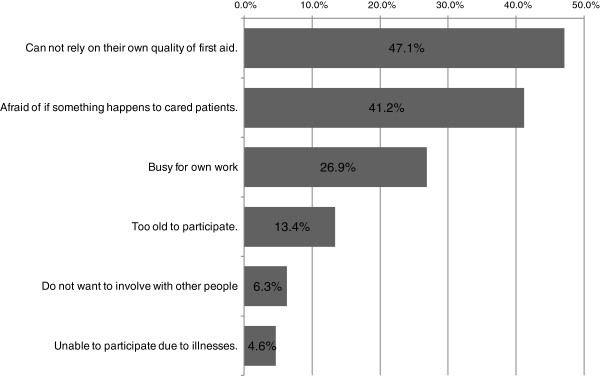
**Individual survey results.** Reason for declining to participate as a CFR in three municipalities in Tochigi prefecture.

The two most common reasons for declining to participate were not being able to rely on their own quality of first aid (47.1%) and being worried about something happening to the patients being cared for, such as death (41.2%) (Figure 1).

**Figure 2:**
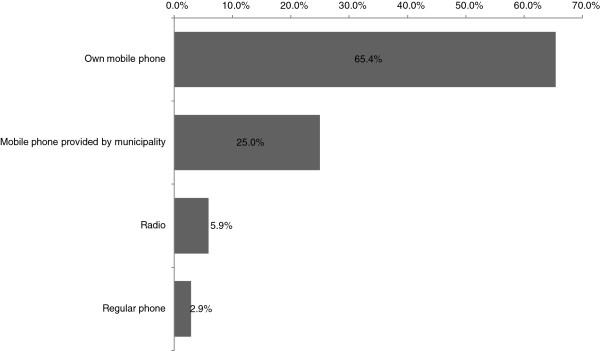
**Individual survey results.** The best communication devices for receiving information about patients.

The best communication device for receiving information about a patient from a dispatch center, according to residents who agreed to participate as a CFR in a remote area, was their own mobile phone (65.4%) for getting response requests from a dispatch center (Figure 2).

### Investigations of preparatory requirements

After the trials, we decided to investigate two preparatory requirements. These were (1) an information delivery system for CFRs since it is essential to minimize the response time of CFRs and (2) budget preparation to maintain their equipment, such as AEDs, for continuation of the CFR operation.

The triage protocols for OHCA patients have already been followed at the dispatch center. Major revision of the protocol was not needed to triage OHCA patients for CFR dispatch. We also found that our pilot area residents were experienced in disaster management because of regular practical training, which includes first aid. Therefore, we did not consider triage protocols and practical training to be serious problems that need to be solved at this time.

We investigated (1) an information delivery system for CFRs and (2) budget preparation individually.

Concerning the information delivery system for CFRs, we found that the current EMS dispatch centers in Japan require major remodeling to add new CFR dispatch functions. Therefore, we added one Internet-accessible computer to dispatch CFRs via e-mail. During our trial, we were able to dispatch a CFR after 119 emergency calls in 1 min 50 s.

Concerning budget preparation, to operate a CFR system, their equipment, such as AEDs, pocket shields or pocket masks, gloves, some kind of communication devices, and jackets to identify them, needs to be prepared by community members themselves. Many Japanese rural areas have community self-government. They collect fees for disaster management. This local disaster management fee might be a budget source for a CFR.

### Development of introduction guidelines

Our individual surveys and pilot trials could identify prerequisites for the introduction of CFR systems. Therefore, using these findings and referring to other countries’ CFR guidelines [6-9], we have discussed what should be prepared and addressed for Japanese people to start CFR systems in their communities. We have developed the introduction guidelines. The table of contents is shown in Table 3.

**Table 3:** Table of contents for CFR introduction guidelines

**Introduction**	
Overview	
CFR introduction criteria	
1.	Criteria
2.	Flow for introduction
3.	Definition of community first responder
Introduction of CFR	
1.	CFR recruitment
2.	Requirement for certification
3.	Responding area
4.	Responding calls
5.	Calls for not responding
6.	Patient care at the scene
7.	CFR call triage
8.	Information delivery for CFR
9.	Responding options
10.	Tasks after ambulance crew arrival
Preparation for CFR introduction	
1.	Flow of preparation
2.	CFR candidate
3.	Cooperation of local EMS and authority
4.	Preparation of CFR equipment
5.	Preparation at dispatch center
6.	Preparation of the budget
7.	CFR education (initial and continuous)
Confidentiality	
Indemnity and insurance	
Liability	
Administration	
Operation after introduced	

### CFR system introduction into the pilot area using the guidelines

After referring to our guidelines, Shioya community held meetings and received training in CPR and AED use from the local EMS. This included practical training that simulated real-life events. All initial costs were covered by our research project. All CFRs were equipped with a pocket mask, identifying jacket, and first aid kit. For communication, the CFR’s own personal phone was used. AEDs were set at four different locations in the town at streets that were easily accessible from most of the CFRs’ homes. The CFRs decided to have their communities maintain their equipment.

Eventually, they were able to start Japan’s first CFR system on 4 November 2012 with 32 participants as CFRs. On 1 January 2013, a CFR responded for the first time, arriving 4 min earlier than the local EMS and performing BLS (Table 4). Although the patient’s rhythm did not require an AED, the CFR system worked efficiently.

**Table 4:** CFR operation on 1 January 2013 at the town of Shioya, Ishikawa, Japan

**H:min:s**	**Event**
6:30:51	119 emergency medical call received at emergency medical dispatch center
6:31:40	Dispatcher suspected out-of-hospital cardiac arrest
6:33:39	The closest ambulance car directed to respond
6:34:25	Fire engine directed to respond for support
6:36:02	Sent patient information e-mail to community first responder
6:42†	Community first responder #1 arrived at the patient home with AED and started CPR
6:43†	Community first responder #2 and #3 arrived at the patient home and attached AED
6:44:35	Community first responder turned on AED
6:45:08	First AED analysis and no shock advised
6:46:43	Fire engine arrived at the patient home and patient care handed over to fire fighter
6:47:29	Ambulance car arrived at the patient home and started care
6:47:44	Second AED analysis and no shock advised
7:04:55	Ambulance care departed for transport

†Time is calculated from the AED activation and the number of CPR cycles performed by the community first responder.

## Discussion

In Japan, the main OHCA patient care comes from ambulance crews. The survival rate is reported to drop dramatically when an ambulance crew takes more than 10 min to arrive and start CPR on the OHCA patient [10], and the use of an AED resulted in 26.5% survival (cardiac origin, witnessed); survival was only 6.3% for non-AED use [10]. In Rochester, Minnesota, use of a first responder system has resulted in a 6.4-min call to shock time, with 46.3% neurologically intact survival (cardiac origin, witnessed, VF) [5]. A similar positive investigation was reported in Piacenza, Italy [2]. Those positive results were achieved because of good responder resources such as police forces and other volunteers; however, most of Japan’s remote areas do not have such resources. To improve the OHCA survival rate in remote areas, local community members must be the first responders to OHCA patients. Since there are many such remote areas in Japan, a CFR system should be introduced in Japan. Although there were many volunteers after the major earthquake on 11 March 2011, a CFR system has not been activated for the purpose of saving OHCA patients.

One of the main reasons for the absence of a CFR system may be related to Japanese administrative history. Japan had a feudalistic system for a long time [11]. Therefore, Japanese people have a tendency to accept what the public administration provides [12]. Currently, Japan’s EMS is heavily dependent on public ambulance services. However, our investigation revealed that 92.5% of rural people agreed to have a CFR system in their communities. This implies that Japanese people may not know how to start volunteering for EMSs such as the CFR program.

Participation as a CFR was a different story. Only 16.7% of people agreed to participate as a CFR in Tochigi prefecture. According to research by the Ministry of Education, 81.9% of people are interested in joining some type of volunteer program [13]. A CFR participant will interact with those facing a life-threatening condition and may encounter an unsuccessful resuscitation. These may be reasons why there is a diminished willingness to participate as a CFR compared to the total number of individuals who claim to have interest in joining a volunteer program. However, we think that the number of more than a few percentages of people who will participate as CFRs is enough to operate a CFR system in remote areas.

Our survey showed that the two top reasons for declining CFR participation were (1) people think that their quality of performance as a CFR may be insufficient and (2) people are fearful of negative patient outcomes. However, we think that these reasons can be managed by (1) providing training to increase responder’s quality of CPR, (2) creating well-organized compensation for CFRs, and (3) providing mental health support.

In Shioya town, to support these residents and to coordinate the necessary management procedures, we found that the existence of a leader with strong support among residents was invaluable. Therefore, creating good leadership among residents may be an important factor when starting a CFR system in a remote area.

Dispatch of CFRs to the scene requires the method that is least time consuming since the CFR is responding to people with life-threatening conditions. In Japan, emergency medical calls are usually controlled by only one call taker and one dispatcher. When they receive a call of suspected cardiac arrest, a dispatcher sends an ambulance and all available EMSs including fire engines. A call taker advises about carrying out the BLS technique over the phone. This shows that the dispatch center personnel have minimal time to dispatch the CFR. Therefore, the dispatch center needs a simple system to provide information to a CFR. Our survey revealed that many people wish to use their own mobile phone to get information from dispatch centers. The saturation level (the number of mobile phones per population) of mobile phones in Japan was 102.67% in 2011 [14]. Therefore, dispatch centers should have a device that can send an e-mail to the responder’s mobile phone. Ideally, a system should be incorporated into current EMS dispatch systems that automatically detects CFR locations and gives the information to the nearest CFRs. However, our CFR system pilot trial revealed that this would incur high costs. We think our simple computer system is reasonably cost effective.

CFR system operation requires budgeting to install and maintain the equipment, such as AEDs and other equipment for continuous CFR system operation. In other countries, CFRs hold fund-raising events and take donations. Since there is no history of CFRs in Japan, people may not be good at such fund-raising activities. However, our investigations found that many community self-governments collect fees regularly for damage restoration from natural disasters such as earthquakes and typhoons. When CFRs become popular in Japan, other fund raising activities could be one of the best resources. However, until then, local disaster management fees could be a temporary funding source for CFR system operations.

When we introduced a CFR system into a pilot area, participating CFR members were worried about their CPR quality, injury compensation, and mental effects such as post-traumatic stress disorder. For these problems, they decided to create (1) a continuing education program to maintain CFR’s quality of CPR every 3 months, (2) a contract with an insurance company to have coverage for injuries and damages, and (3) an agreement with the local government that CFRs have the right to use the mental health services of the local EMS whenever they feel the need for counseling. For information delivery from dispatch centers, each CFR member had a mobile phone with e-mail capability. Therefore, the dispatch center just added one Internet-accessible computer for CFR dispatching. All these preparations led to the successful introduction and operation of the CFR system.

## Conclusions

Although there had been no CFR operations in Japan until now, by providing information about a CFR system to the community and preparing several infrastructural elements, it was possible to introduce and operate a CFR system in Japan. The ultimate goal of introducing the CFR system into Japan is to save cardiac arrest patients in remote areas. Further studies are needed to spread information about CFRs throughout Japan and evaluate the effects on clinical outcomes.

## Abbreviations

OHCA: Out-of-hospital cardiac arrest; CFR: Community first responder; EMS: Emergency medical service; CPR: Cardiopulmonary resuscitation; AED: Automated external defibrillator; VF: Ventricular fibrillation.

## Competing interests

The authors declare that there are no competing interests.

## Authors’ contributions

YT wrote the entire article and is the project manager for the individual survey. MS is the chief researcher for this project. KY is the project supervisor. CY is the project manager for the dispatch system. KK and YY are the project managers for guideline development. AK coordinated the individual survey and pilot trials. HM coordinated the introduction of the system into Shioya town. All authors read and approved the final manuscript.
